# La_2_Sn_2_O_7_/g-C_3_N_4_ nanocomposites: Rapid and green sonochemical fabrication and photo-degradation performance for removal of dye contaminations

**DOI:** 10.1016/j.ultsonch.2021.105678

**Published:** 2021-07-24

**Authors:** Zeinab Talebzadeh, Maryam Masjedi-Arani, Omid Amiri, Masoud Salavati-Niasari

**Affiliations:** a Institute of Nano Science and Nano Technology, University of Kashan, Kashan, P. O. Box.87317-51167, Iran; b Faculty of Chemistry, Razi University, Kermanshah 6714414971, Iran; c Department of Chemistry, College of Science, University of Raparin, Rania, Kurdistan Region, Iraq

**Keywords:** Nanostructure, Ultrasonic irradiation, Graphitic carbon nitrides, La_2_Sn_2_O_7_, Photocatalyst

## Abstract

The deficiency of drinking water sources has become a serious crisis for the future of the world that the photocatalytic process is one of the most favorable methods for removal of artificial dyes and poisonous organic impurities. In the present study, rapid ultrasonic treatment was performed to obtain La_2_Sn_2_O_7_/Graphitic carbon nitrides (LSO/CN) nanocomposites with advanced photo-catalytic performance. Broccoli extract was utilized as a natural surfactant with active surface groups to control nucleation and growth of formed crystals with the creation of spatial barriers around the cations, and finally prevent nano-product agglomeration. Changing experimental parameters in synthesis reaction in turn offers a virtuous control over the nano-products size and shape. The shape and size distribution of particles was considered *via* diverse characterization techniques of microscopic and spectroscopic. The photocatalytic behaviors along with a kinetic study of the nanoparticles were examined by elimination and degradation of different artificial dyes under the UV waves. Effect of particle size, weight ratio of LSO:CN, type of dye, scavenger kind, dye and catalyst loading was designated on altering proficiency of nano-catalyst function. Also, the probable mechanism of removal dye by photocatalytic function was studied.

## Graphical abstract

**Figure:**
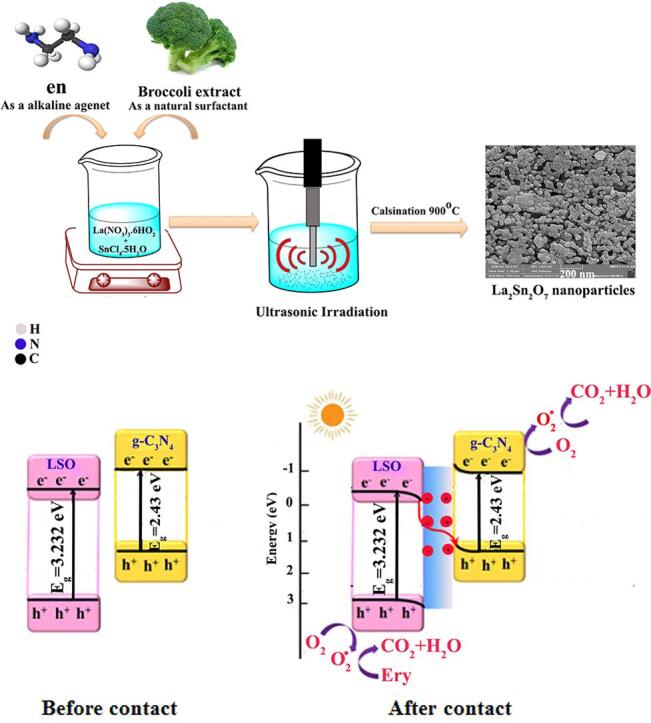

## 1 Introduction

Nowadays, the deficiency of drinking water sources has become a serious crisis for the future of the world due to the existence of numerous artificial dyes and poisonous organic impurities in aqueous environments. There are many methods to remove artificial dyes from drinking water such as nanofiltration [1], adsorbent [2], biosorption [3] and the photocatalytic process [4], [5]. Among them, the photocatalytic activity received more attention because of its privileges of low energy consumption, high stability, environmental and economical friendly [6], [7], [8], [9]. The mechanism of photocatalytic process is described in the following path: (1) the absorption of photons with energy ≥ the bandgap of nano-photocatalyst, (2) photoexcited electrons create at conduction band and the same amount of positive holes at valence band, (3) The various oxidants of OH^•^, O_2_^•^ and H_2_O_2_ could proficiently oxidize dye compounds into harmless combinations of CO_2_ and water [10], [11], [12].

Numerous efforts have been prepared in the field of produce various forms of photocatalyst such as graphene-based nanocomposite [13], [14], binary oxides [10], [15] and ternary nano-photocatalyst [16]. La_2_Sn_2_O_7_ Pyrochlore-type oxide as a semiconductor nano-oxide has an appropriate performance in diverse applications such as photocatalysis [17], energy storage [18], [19] and catalysis [20], [21]. Owning to the intense efficacy of fabrication approaches on the form and dimension of nanoproducts, the preparation path of pyrochlore La_2_Sn_2_O_7_ nano samples is significant. La_2_Sn_2_O_7_ nano-sized structures were created *via* several chemical techniques [17], [22], [23]. Despite the extensive researches carried out on nano-photocatalyst synthesis and performance, there is still an essential requirement to suggest a beneficial nano-scale sample through a low-cost, fast and eco-friendly way. Recently, green chemistry-based methods have been noticed to the creation of diverse nanostructures due to their non-hazardous and safe features to the environment [24], [25], [26]. The avail of Broccoli extract to synthesize the nanosized La_2_Sn_2_O_7_ structures has not yet been considered. This work offers a rapid eco-friendly sonochemical method to fabrication of La_2_Sn_2_O_7_ nanostructures as Uv-light-sensitive photocatalyst with the aid of Broccoli extract, for the first time. Ultrasonic irradiations by increasing reaction rate owning to create of high temperature and pressure in a liquid medium are led to chemical reaction accomplishment in a short time [27]. The sonochemical technique is more favorable in terms of low cost, simply control the form and particle dimension through changing of ultrasonic time and power, low processing temperature, simplicity and potential for large-scale production [28], [29]. Compared with other chemical approaches, green sonochemistry has the benefits of quicker reaction rate, lower particle size, more uniformity and purity for the synthesis of nano-scaled powder [30]. Broccoli is rich in phytochemical components including glucosinolates and polyphenols. These agents can conjugate to created nuclei, control their growth and finally create favorable nano-sized samples [31], [32]. Therefore, broccoli extract was utilized as both green surfactant and alkaline agent [33]. Despite many research about La_2_Sn_2_O_7_ structures, the effect of sonochemical reaction condition using the broccoli extract as a green surfactant on their photocatalytic properties of remain unclear. In the present work, it is aimed at considering nano-sized La_2_Sn_2_O_7_ structures synthesized by ultrasonic irradiation in presence of green broccoli surfactant. Graphitic carbon nitride (g-C_3_N_4_) polymeric component was selected to the formation of La_2_Sn_2_O_7_/CN nanocomposite because of suitable energy gap (2.7 eV), high surface area and cheap synthesis route as well as finally, advanced photocatalytic activity [34]. Numerous researches illustrate that using g-C_3_N_4_ has a favorable impact on photocatalytic efficiency [35], [36]. In this work, La_2_Sn_2_O_7_/ g-C_3_N_4_ nanocomposites were fabricated via green and rapid ultrasonic technique in presence of broccoli extract natural surfactant. After characterization of obtained nano-products in various experimental circumstances, photo-degradation efficiency of them was evaluated in several experimental tests such as particle size, weight ratio of LSO:CN, type of dye, scavenger kind, dye and catalyst loading. Moreover, the photo-driven degradation mechanism of erythrosine dye by LSO/CN nanocomposite was studied.

## 2 Experimental

### 2.1 Materials and physical measurements

All the chemical reagents for the synthesis of La_2_Sn_2_O_7_/ g-C_3_N_4_ nanocomposites such as La(NO_3_)_3_·6H_2_O (99.99%), SnCl_4_·5H_2_O (98%) and melamine were commercially available and employed without further purification. A multiwave ultrasonic generator (MPI Ultrasonics; welding, 1000 W, 20 KHz, Switzerland), immersed directly in the reaction solution. X-ray diffraction (XRD) patterns were recorded by a Philips-X’pertpro, X-ray diffractometer using Ni-filtered Cu Ka radiation. Fourier transform infrared (FT-IR) spectra were recorded on Nicolet Magna- 550 spectrometer in KBr pellets. The electronic spectrum of the sample was taken on Perkin–Elmer LS-55 luminescence spectrometer. Scanning electron microscopy (SEM) images were obtained on LEO-1455VP equipped with an energy dispersive X-ray spectroscopy. The EDX analysis with 20 kV accelerated voltage was done. Transmission electron microscopy (TEM) image was obtained on a Philips EM208 transmission electron microscope with an accelerating voltage of 200 kV.

### 2.2 Preparation of broccoli extract

An appropriate amounts of fresh broccoli leaves were carefully washed *via* distilled water and air-dried. Then, the clean leaves were crushed in the food processor and filtered *via* filter paper. The obtained broccoli extract was kept in a refrigerator for further use.

### 2.3 Sonochemical fabrication of La_2_Sn_2_O_7_ nano products

**Scheme 1:**
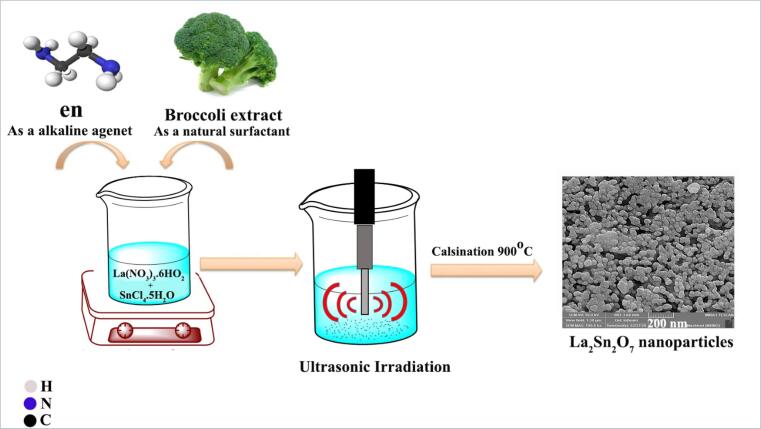
Schematic model for sonochemistry preparation of LSO nanoparticles by natural surfactant.

La(NO_3_)_3_·6H_2_O (99.99%) and SnCl_4_·5H_2_O (98%) salts were purchased from Sigma-Aldrich Company and weighed based on stoichiometric ratios (1:1) and dissolved in deionized water, separately. Then, two solutions were added to each other. Broccoli extract was utilized as a natural surfactant. Meanwhile, NH_3_ was added to the cationic solution dropwise till set the pH to 11. Afterwards, A multiwave ultrasonic generator (MPI Ultrasonics; welding, 1000 W, 20 kHz, Switzerland), immersed directly in the reaction solution for 15 min. The attained products were centrifuged, dried and calcined at 900 °C for 5 h. In order to reach favorable dimension and morphology of nano-scaled structures for enhanced photocatalytic performance, the effect of calcination conditions, ultrasonic time and type of alkaline agent was evaluated that has been exemplified in Table 1. Also, a blank test without Broccoli surfactant was carried out. The obtained nanocomponents were considered through various physical analyses. The schematic plan of La_2_Sn_2_O_7_ nanoparticle creation by sonochemical route has been displayed in Scheme 1.

**Table 1:** The carried out experimental reactions.

Sample lable	Calcination condition	Ultrasonic irradiation time (min)	Alkaline agent	Natural surfactant	Crystalline size (nm)
LSO1	800 °C, 3 h	15	NH_3_	–	–
LSO2	900 °C, 3 h	15	NH_3_	–	–
LSO3	900 °C, 5 h	15	NH_3_	–	22.4
LSO4	900 °C, 5 h	–	NH_3_	–	25.7
LSO5	900 °C, 5 h	15	en	–	23.9
LSO6	900 °C, 5 h	15	en	Broccoli extract	30.3
LSO7	900 °C, 5 h	30	en	Broccoli extract	25.8

### 2.4 Preparation of g-C_3_N_4_/La_2_Sn_2_O_7_ nanocomposites

The optimized La_2_Sn_2_O_7_ nano-samples in terms of size and shape along with melamine were dispersed in ethanol in an ultrasonic bath for 30 min. then, the centrifuged precipitates were dried and heated at 500 °C for 4 h. The g-C_3_N_4_/LSO nanocomposites prepared in various weight ratios have been listed in Table 1.

### 2.5 Photocatalytic tests

The photodegradation process was accomplished in a home-produced glass reactor set containing 100 mL aqueous solutions of 10 ppm of erythrosine and methyl violet. For every reaction, 10 mg La_2_Sn_2_O_7_ nano-structures were dispersed in a dye solution. The achieved suspensions were stirred at room temperature and kept in dark circumstances for 30 min. To end, the organization set was irradiated under a UV lamp (Osram ULTRA-VITALUX 300 W) consisting of UVA (λ = 320 to 400 nm) and UVB (λ = 290–320 nm).

## 3 Result and discussion

### 3.1 X-ray diffraction investigations

**Fig. 1:**
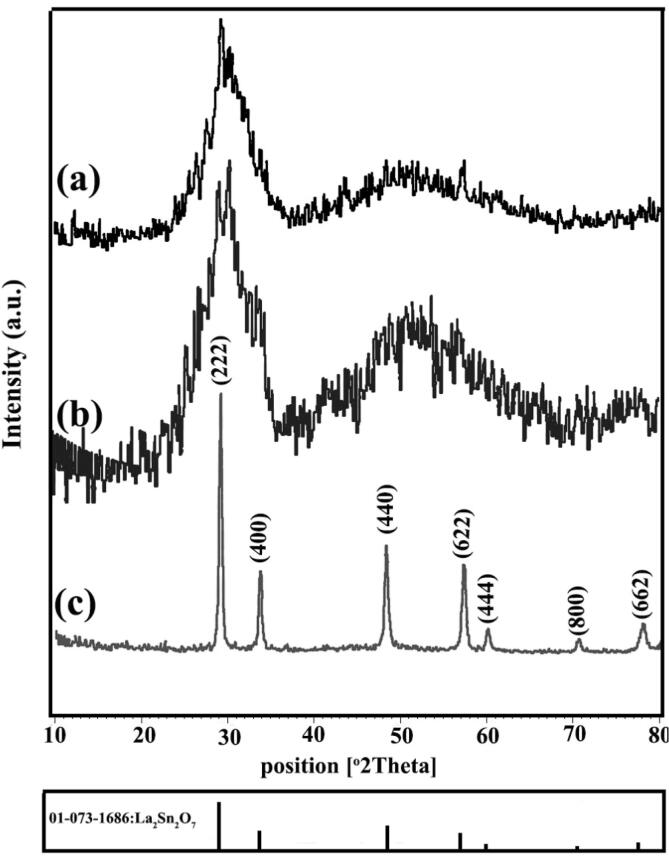
XRD patterns of La_2_Sn_2_O_7_ nano-samples prepared in different calcination circumstances (a) LSO1, (b) LSO2 and (c) LSO3.

**Fig. 2:**
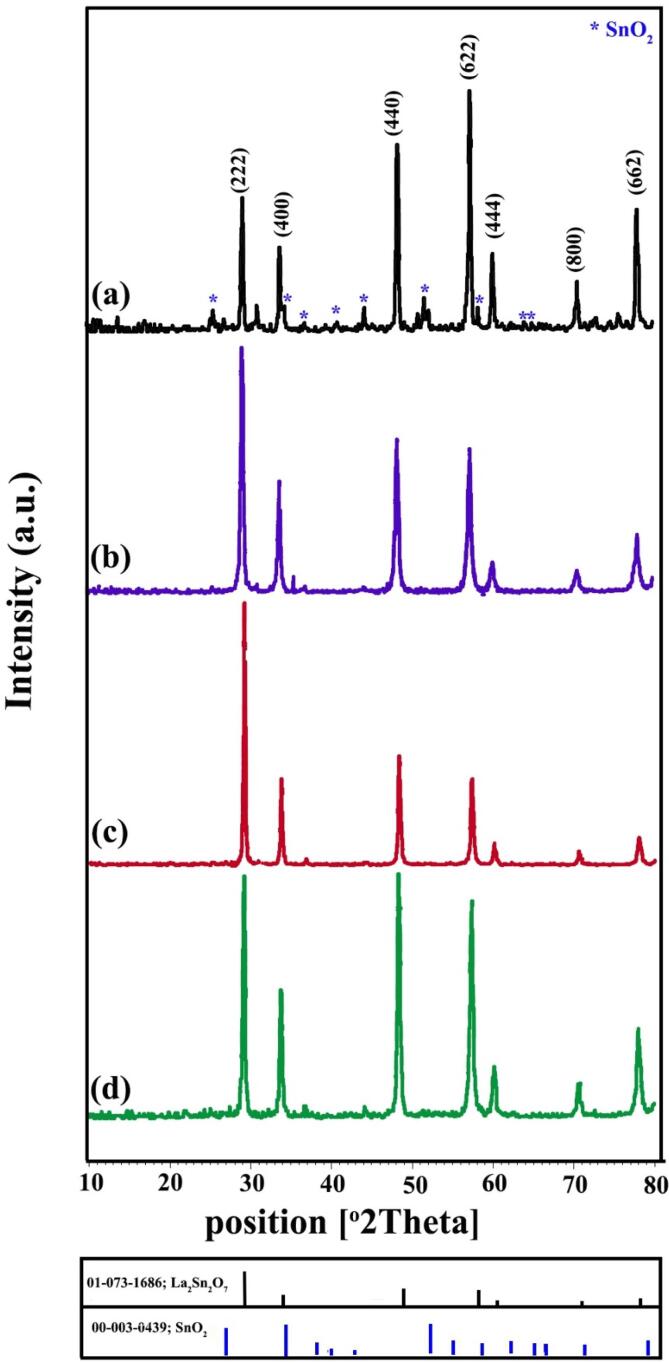
XRD patterns of La_2_Sn_2_O_7_ nano-samples prepared in different experimental circumstances (a) LSO4, (b) LSO5, (c) LSO6 and (d) LSO7.

X-ray diffractograms of La-Sn-O sample prepared through sonochemical route in calcination condition of 800 °C for 3 h (LSO1) have been presented in Fig. 1a. This time and temperature of heat treatment are not sufficient for the preparation of La_2_Sn_2_O_7_ nano-structures. By increasing temperature to 900 °C (LSO2), the peaks are still presented in non-crystallized form (Fig. 1b), but pure cubic La_2_Sn_2_O_7_ structures (JCPDS No = 73–1686) were created at 900 °C for 5 h that have been illustrated in Fig. 1c (LSO3). It was concluded that a heat process of 900 °C for 5 h is essential for the fabrication of La_2_Sn_2_O_7_ nanocrystals. Cubic La_2_Sn_2_O_7_ structures are including four main peaks at 2 that of 28.88, 33.47, 48.06 and 57.04 related to (2 2 2), (4 0 0), (4 4 0) and (6 2 2) plans. The XRD pattern of La_2_Sn_2_O_7_ crystals prepared in blank condition without ultrasonic irradiation has been demonstrated in Fig. 2a (LSO4). By using the precipitation route, La_2_Sn_2_O_7_ powder along with little amount impurity of SnO_2_ (JCPDS No = 03–0439) were formed. To the investigation of alkaline agent, ethylene diamine (en) was utilized instead of ammonia (Fig. 2b). The en has a lower release rate of hydroxide anion than NH_3_. In the presence of en, pure cubic lanthanum tin oxide nano-powder was fabricated (LSO5). In order to formation of structures with smaller dimensions, the presence of surfactant is vital. Fig. 2c represents XRD diffractogram of La_2_Sn_2_O_7_ nano samples in existing of broccoli extract as a natural surfactant (LSO6) that indexed to pure La_2_Sn_2_O_7_ nanostructures (JCPDS No = 73–1686). Moreover, one specimen was synthesized with an increasing ultrasonic irradiation time of 30 min (LSO7) that has been indicated in Fig. 2d. As seen in Fig. 2d, the peaks intensity of La_2_Sn_2_O_7_ nanostructures has increased. The crystalline size of La-Sn-O nano-grains was calculated by the Scherer equation [37] and listed in Table 1.

### 3.2 Morphology investigation (SEM & TEM)

**Fig. 3:**
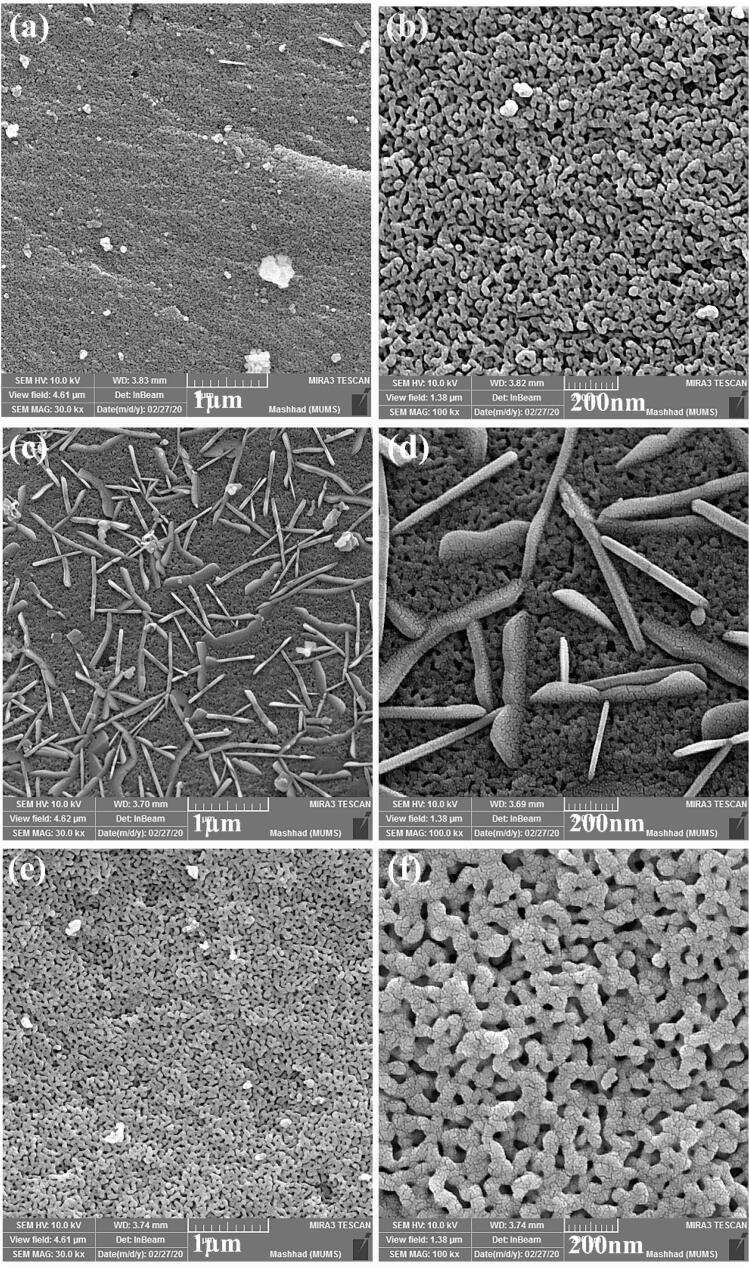
SEM images of La_2_Sn_2_O_7_ nano-samples prepared in different calcination circumstances (a, b) LSO1, (c, d) LSO2 and (e, f) LSO3.

Fig. 3 indicates SEM images of La_2_Sn_2_O_7_ nanostructures fabricated in various times and temperatures of heat treatment. Interconnected particles were formed in 800 °C for 3 h (Fig. 3a, b) that by increasing temperature to 900 °C for 3 h (Fig. 3c, d), nanorods with the size of 30 nm in diameter and 500 nm in length were created besides aggregated particles. With the intensification of time to 5 h, regular particles were synthesized (Fig. 3e, f).

**Fig. 4:**
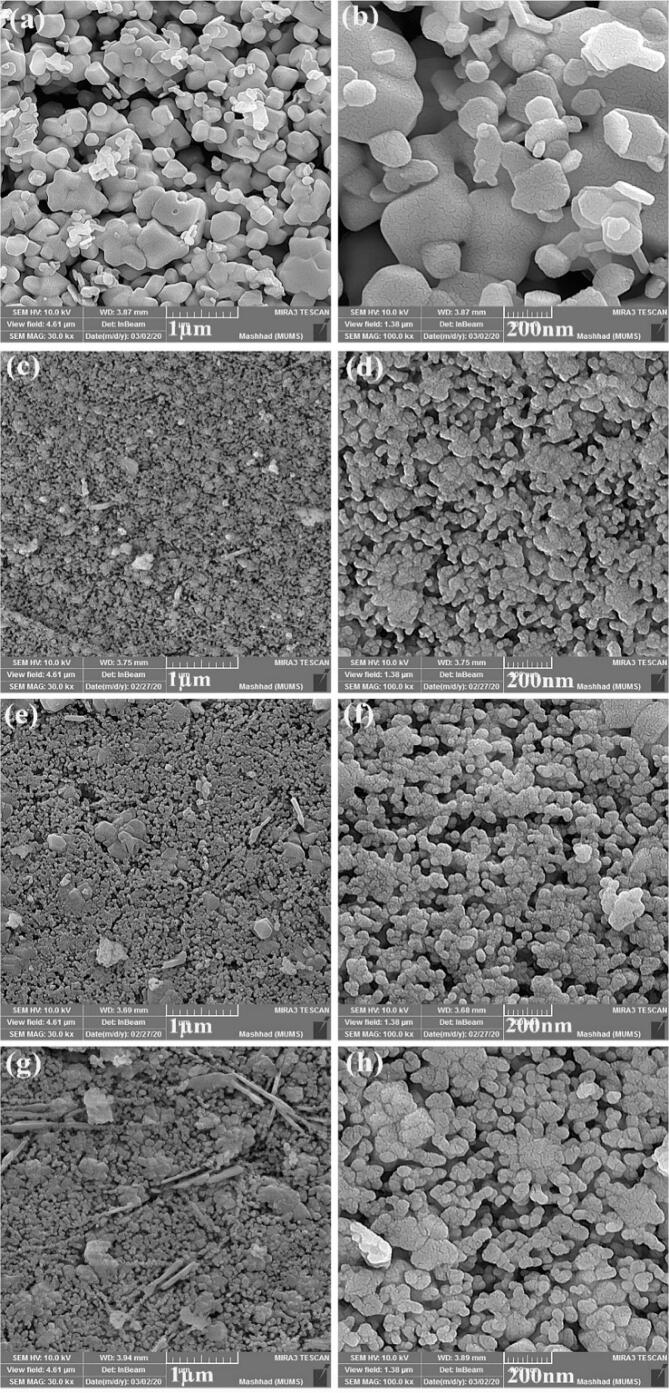
SEM images of La_2_Sn_2_O_7_ nano-samples prepared in different experimental circumstances (a, b) LSO4, (c, d) LSO5, (e, f) LSO6 and (g, h) LSO7.

La_2_Sn_2_O_7_ structures prepared through precipitation technique without ultrasonic irradiation have large and irregular size that shown in Fig. 4a, b. ultrasonic waves help to formation uniform fine particles with accelerating chemical reaction. To investigate of precipitating operator on the final properties of LSO products, the ethylene diamine was applied instead of ammonia. As observed in Fig. 4c, d, uniform particles were synthesized with the size range of 20–40 nm. The en has a lower release rate of hydroxide anions (OH^−^) than NH_3_ and finally, helps to control of nucleation and growth of nanoparticles. Fig. 4e, f present SEM images of La_2_Sn_2_O_7_ nanostructures prepared in presence of Broccoli extract. Spherical nanoparticles formed have homogeneous size and shape. Broccoli was utilized as both green surfactant and alkaline agent including active glucosinolate and polyphenol groups that can conjugate to created nuclei, control their growth and finally create favorable nano-sized samples [31], [32]. The influence of sonochemical time was evaluated on the appearance features of La_2_Sn_2_O_7_ nano-products. By growing reaction time of ultrasonic to 30 min, larger particles were created with a range size of 20–100 nm (Fig. 4g, h) and reaction time of 15 min was designated as an ideal time.

**Fig. 5:**
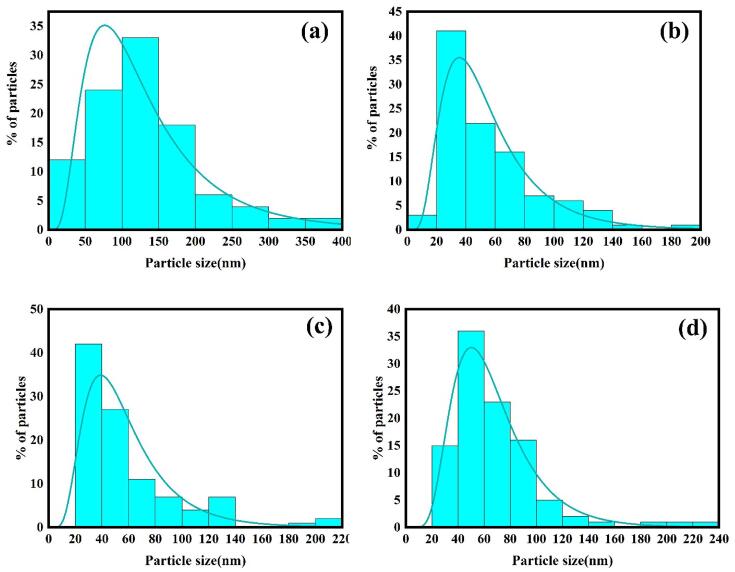
Particle size distribution curves of La_2_Sn_2_O_7_ nano-samples prepared in different experimental circumstances (a) LSO4, (b) LSO5, (c) LSO6 and (d) LSO7.

Particle size distribution diagrams of La_2_Sn_2_O_7_ nanostructures obtained in different circumstances (LSO4, LSO5, LSO6 and LSO7) have been demonstrated in Fig. 5 that calculated by Digimizer software. With evaluating of particle size distribution diagrams was concluded that the lowest size distribution is related to La_2_Sn_2_O_7_ nanostructures prepared via broccoli extract and 15 min ultrasonic irradiation (LSO6).

**Fig. 6:**
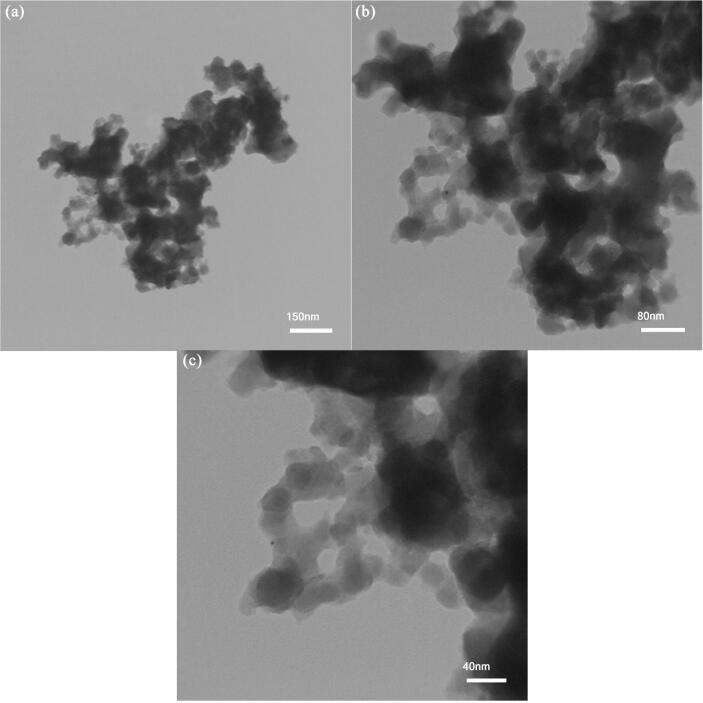
TEM images of LSO6 nanoparticles in different scales.

Fig. 6 illustrates TEM pictures of optimized La_2_Sn_2_O_7_ nanoparticles fabricated via sonochemical route and broccoli natural surfactant (LSO6). Spherical-shaped nanoparticles have a size about 15–50 nm.

### 3.3 Ultrasonic formation mechanism

La_2_Sn_2_O_7_ nano-products with optimized features are created through the simultaneous outcomes of ultrasonic irradiation and broccoli as a natural surfactant. Appropriate structures in the nano-sized form are produced *via* the generated cavitation with ultrasound waves. Excessive temperatures and pressures are created with huge energies according to hot-spot theory and make active components of radicals [28]. The development of preparation mechanism of samples *via* the sonochemical method stated as below:

H_2_O → H^•^ + OH^•^

H^•^ + H^•^ → H_2_

OH^•^ + OH^•^ → H_2_O_2_

NH_3_ (or en) + H_2_O + H^•^ → NH_4_^+^ (en-H^+^) + OH^−^

Sn^4+^ + OH^−^ → Sn(OH)_4_

La^3+^ + Sn(OH)_4_ + OH^−^ → La_2_Sn_2_O_7_ nanoparticles

### 3.4 Optical features

**Fig. 7:**
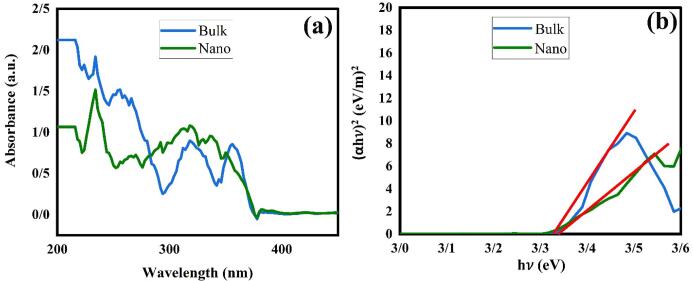
(a) UV–Vis diffuse absorption spectra and (b) linear portion of plots of (αhν)^2^ against (hν) of bulk and nano LSO products.

UV–vis absorption mode was utilized to evident the optical features and energy structure in semiconductor materials. The UV–Vis outcomes for La_2_Sn_2_O_7_ nano (LSO6) and bulk (LSO4) particles were exposed in Fig. 7a, b. The absorption peaks for both samples are around 200–400 nm. The band gap of La_2_Sn_2_O_7_ nano and bulk specimens was gauged by Tauc’s equation [38] about 3.35 and 3.30 eV, respectively.

**Fig. 8:**
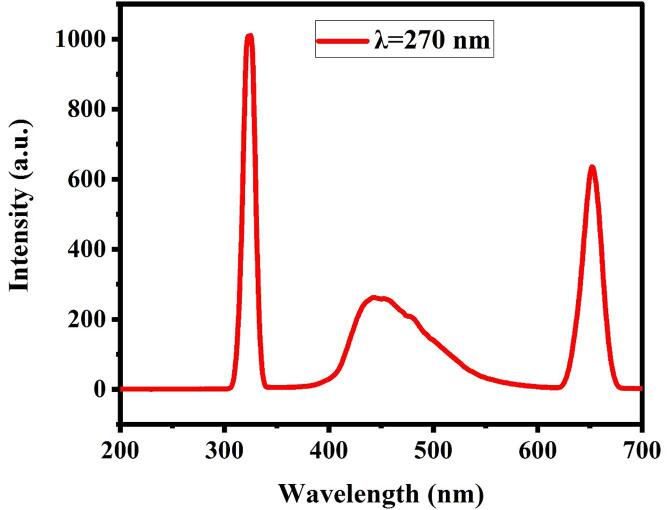
Room-temperature emission spectrum of optimized La_2_Sn_2_O_7_ nano crystals (LSO6).

The luminescence characteristic of La_2_Sn_2_O_7_ nano crystals prepared *via* sonochemical route in optimized circumstances has been illustrated in Fig. 8 under the excitation of 270 nm. As shown in Fig. 8, three peaks exist at 320, 450 and 650 nm. In the La_2_Sn_2_O_7_ nanoparticle, the La and Sn cations with inversion symmetry are coordinated to eight and six oxygen, respectively in a geometry only slightly distorted from a regular octahedron [39].

### 3.5 Characterization of La_2_Sn_2_O_7_/g-C_3_N_4_ (LSO/CN) nanocomposites

**Fig. 9:**
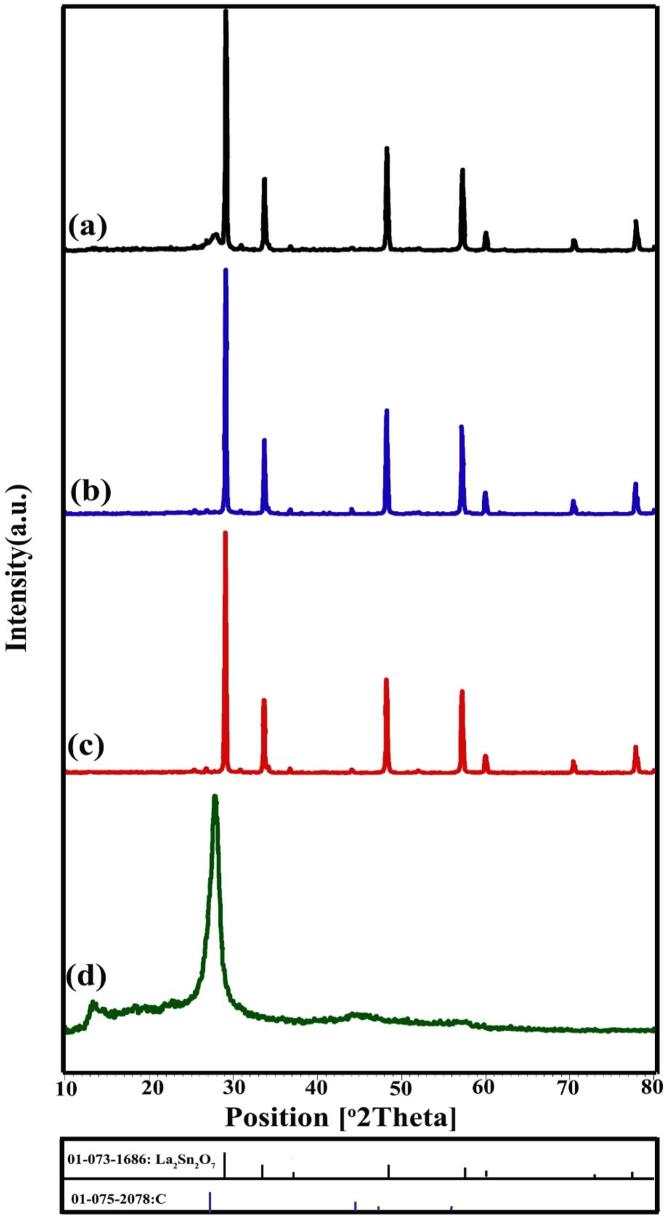
XRD patterns of LSO/CN nanocomposites prepared in various weight ratios of LSO/CN (a) 30:70, (b) 50:50, (c) 70:30 and (d) pristine g-C_3_N_4_.

Fig. 9a-c display XRD patterns of LSO/CN nanocomposites prepared in various weight ratios of LSO/CN 30:70, 50:50 and 70:30, respectively. All diffractograms confirm the presence of La_2_Sn_2_O_7_ nanocrystals. Owing to the small intensity of CN peak versus great intensity of La_2_Sn_2_O_7_ peaks, existing of LSO is not significantly observed. In Fig. 9a, a small peak in 2θ about 28° is seen because of high weight ratios of LSO/CN 30:70. Fig. 9d present the XRD pattern of pristine g-C_3_N_4_ component (JCPDS No. 75-2078).

**Fig. 10:**
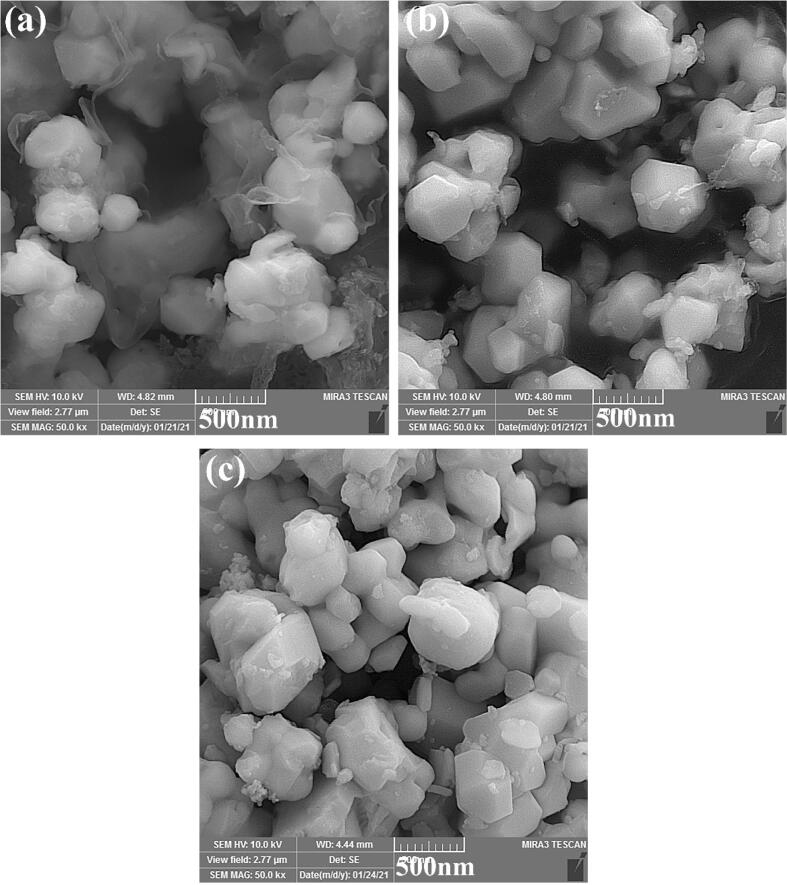
SEM images of g-C_3_N_4_/La_2_Sn_2_O_7_ nanocomposites prepared in various weight ratios of LSO/CN (a) 30:70, (b) 50:50 and (c) 70:30.

The SEM images of La_2_Sn_2_O_7_/g-C_3_N_4_ (LSO/CN) nanocomposites obtained in different LSO:CN weight ratios of 30:70, 50:50 and 70:30 have been specified in Fig. 10a-c. The homogeneous distribution of LSO nanoparticles in ultra-thin sheets of g-C_3_N_4_ is confirmed. Moreover, more amount CN sheets are observed in Fig. 10a.

**Fig. 11:**
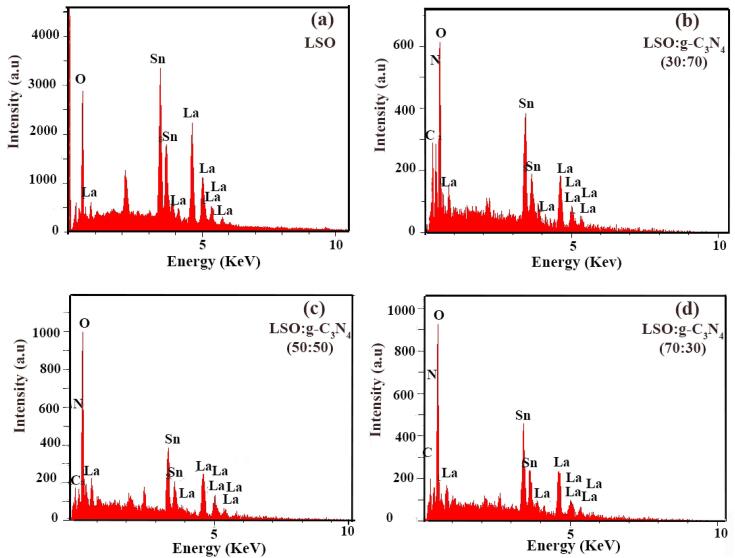
EDX spectra of (a) pure LSO, different LSO/CN weight ratios of (b) 30:70, (c) 50:50 and (d) 70:30.

The results of EDX analyses of pure LSO nanoparticles and divers LSO/CN nanocomposites have been represented in Fig. 11a-d, respectively. In Fig. 11a, the existence of La, Sn and O lines confirm the formation of pure La_2_Sn_2_O_7_. Also, in Fig. 11b-d presence of C and N peaks along with La, Sn and O lines approve the combination of LSO and CN in nanocomposites. Moreover, the intensity of C and N peaks is higher in LSO:CN weight ratio of 30:70.

### 3.6 BET analysis

**Fig. 12:**
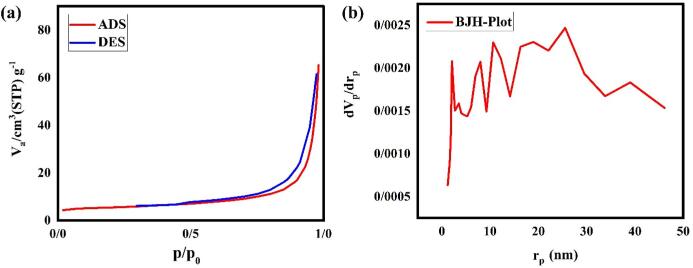
(a) N_2_ adsorption/desorption isotherms and (b) pore size distribution of LSO/CN nanocomposite with weight ratio of 30:70.

The nitrogen adsorption–desorption isotherms and pore size distribution curves of LSO/CN nanocomposite with a weight ratio of 30:70 have been revealed in Fig. 12a, b. The hysteresis type of nanocomposite is related to aggregate particles with slit-like pores with broad size distribution (type-IV and H3) [40]. The attained pore volume and average pore size for LSO/CN nanocomposite in ideal circumstances are 4.50 cm^3^g^−1^ and 20.28 nm, respectively. Furthermore, the specific surface area evaluates 19.92 m^2^g^−1^. By evaluating obtained BET results, LSO/CN nanocomposite with a weight ratio of 30:70 is a favorable applicant for photocatalysis systems.

### 3.7 Photocatalytic degradation efficiency and kinetic studying

**Fig. 13:**
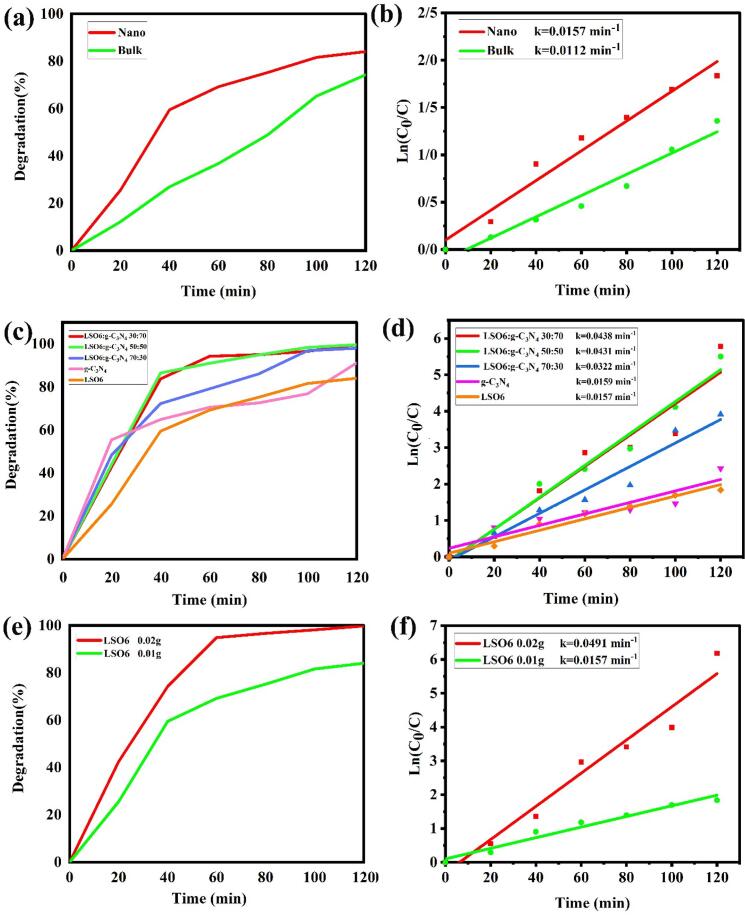
(a), (c), (e) photocatalytic behaviors and (b), (d) and (f) plots of ln(C/C_0_) vs time of various types of LSO nanostructures in diverse photocatalytic experimental circumstances for removal of erythrosine dye.

Due to suitable electronic band gap and high surface area properties of LSO nanoparticles and LSO/CN nanocomposites, the photocatalytic activity of nanostructures was evaluated under UV irradiation for degradation of water pollutants. The impact of various parameters of photocatalytic degradation was investigated in order to achieve the highest degradation percentage. The factors of catalyst type, different weight ratios of LSO:CN, nano-catalyst amount, dye type, dye concentration and different additives as the scavengers were considered. Fig. 13a indicates the photocatalytic performance of LSO nanoparticles prepared via ultrasonic irradiation and LSO bulk structures synthesized without ultrasonic irradiation. The details and results of the degradation efficiency of erythrosine dye under UV light have been presented in Fig. 13. As illustrated in Fig. 13a, degradation efficiency of LSO nanostructures (LSO6) is more than LSO bulk structures (LSO4) that is related to the higher surface area of nano into the bulk. The degradation efficiency of products is calculated as follows (Eq. (1)):

(1) $$\text{degradation}\;efficiency\left(\eta \right)\%=\frac{\left(A_{0}-A_{\text{t}}\right)}{{\text{A}}_{0}}\times 100$$

Which A_0_ and A_t_ are absorbance amounts in times of start and t min. According to Eq. (1), the degradation percentage of nanoparticles and bulk products was obtained 84 and 75 % after 120 min. Also, in order to consider kinetic properties of samples according to Langmuir–Hinshelwood mechanism, the promising reaction rate coefficients can be gained from Eq. (2):

(2) $$\ln\frac{C_{0}}{C_{t}}=kt$$

Which C_0_ and C_t_ are dye concentration in times of start and t min and k is the pseudo-1st-order rate coefficient (min^−1^) [41]. According to linear dependences of ln(C_0_/C_t_) versus reaction time, the 1st-order rate constant k has been attained. As observed in Fig. 13b, the rate constant of k for nanoparticles is more than bulk structures that confirm catalytic degradation of erythrosine dye in presence of LSO nanoparticles is carried out with higher speed. As perceived in Fig. 13c, it is clear that the photo-degradation performance of LSO:CN nanocomposites is more than pristine La_2_Sn_2_O_7_ (LSO6) and g-C_3_N_4_ components. Also, by increasing g-C_3_N_4_ amount in nanocomposite, the rate constant of k rise (Fig. 13d). The cause of advanced photocatalytic behavior of hybrid LSO/CN nanocomposite can be related to the accommodation of energy gaps and electronic bands of LSO and CN specimens and finally, formation of perfect electron migration routes, fast e^−^-h^+^ separation and carrying [42], [43].

Fig. 13e, f exhibit the results of photo-degradation of LSO6 nanocatalyst in different amounts of 0.02 and 0.01 g. It is obvious that photocatalytic activity is completely performed in presence of more amount of nanocatalyst (0.02 g) with speed about four times faster (k = 0.0491 min^−1^) than 0.01 g LSO6 nanocatalyst (k = 0.0157 min^−1^).

**Fig. 14:**
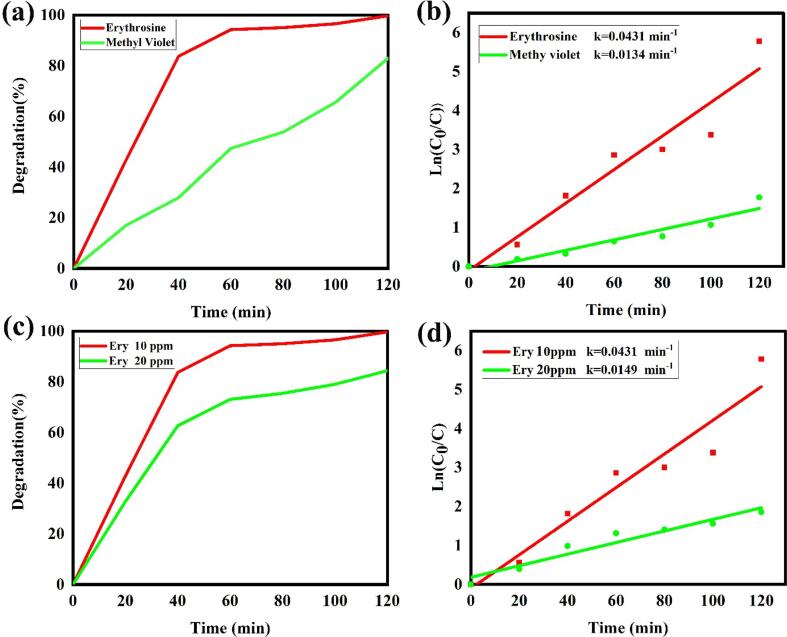
Photocatalytic performance and plots of ln(C/C_0_) vs time of LSO nano-products for different (a), (b) dye solutions, (c) and (d) dye concentrations.

The photocatalytic activity of LSO6 nanocatalyst was evaluated for degradation of anionic erythrosine and cationic methyl violet dyes to acquire developed efficiency. As indicated in Fig. 14a, b, the behavior of La_2_Sn_2_O_7_ nanoparticles for degradation of erythrosine as an anionic dye is better than methyl violet as a cationic dye and photocatalytic efficiency was reported 100 and 82 %, respectively. Also, the degradation speed of erythrosine is higher than methyl violet dye.

Fig. 14c, d compare the photocatalytic performance of LSO6 nanocatalyst in different erythrosine dye concentrations of 10 and 20 ppm. The outcomes exhibit optimized La_2_Sn_2_O_7_ nanoparticles have better function in lower dye concentrations.

**Fig. 15:**
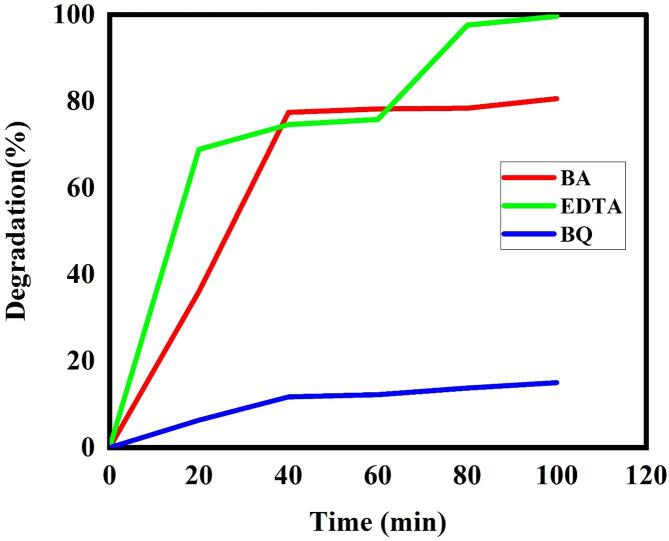
Degradation percentage vs time of LSO nano-products in presence of three types of scavenger of benzoic acid, EDTA and Benzoquinone.

Fig. 15 displays the photo-degradation percentage of LSO6 nanocatalyst in presence of three different scavenger agents of Ethylenediaminetetraacetic acid (EDTA), Benzoic acid (BA) and benzoquinone (BQ) to trap h^+^, ^•^OH and ^•^O_2_^−^ active specimens [44], respectively for more realization of photo-degradation mechanism of erythrosine dye by UV irradiation. As illustrated, the photo-degradation efficiency of erythrosine dye by La_2_Sn_2_O_7_ nano-catalyst has noticeably reduced in presence of BQ as a ^•^O_2_^−^ scavenger. It is concluded that ^•^O_2_^−^ active component has the most contribution in removal dye pollutant of water. However, according to Fig. 15 and a slight decrease in photocatalytic efficiency, ^•^OH also plays little role in destruction of toxic dye. The mechanism of erythrosine contamination via optimized nanocatalyst by superoxide anion and hydroxyl radicals has been summarized in follows:

LSO nano-catalyst + hν → LSO nano-catalyst* (e^−^_CB_ + h^+^_VB_)

O_2_ + e^−^ → ^•^O_2_^−^

^•^O_2_^−^ + e^−^ + 2H^+^ → H_2_O_2_

H_2_O_2_ + e^−^ → OH^−^ + ^•^OH

h^+^ + H_2_O → ^•^OH + H^+^

Erythrosine dye + (^•^O_2_^−^ + ^•^OH active agents) → product + H_2_O + CO_2_

**Scheme 2:**
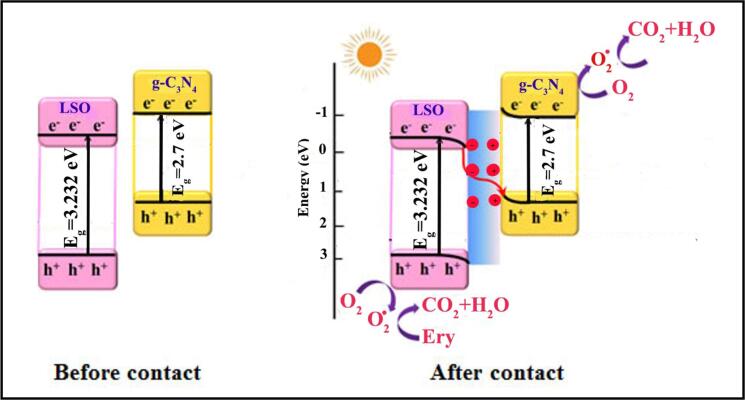
Photo-degradation mechanism of LSO/CN nanocomposite.

The schematic design of photo-degradation mechanism of LSO/CN nanocomposite has been illustrated in Scheme. 2.

## 4 Conclusions

In summary, we were productively designed binary La_2_Sn_2_O_7_/g-C_3_N_4_ nanocomposites through ultrasonic waves with distinctive structural and optical features for photocatalytic activity under UV irradiations for elimination of drinking water pollutants. Effect of particles size, weight ratio of LSO:CN, type of dye, scavenger kind, dye and catalyst loading was designated on altering proficiency of nano-catalyst function. As a result, La_2_Sn_2_O_7_/g-C_3_N_4_ nanocomposites with 30% La_2_Sn_2_O_7_ nanoparticle (ƞ=99%) have better efficiency than pristine La_2_Sn_2_O_7_ nanoparticle (ƞ=72%) and g-C_3_N_4_ specimen (ƞ=91%). Moreover, the photocatalytic activity is completely performed in presence of more amount of nanocatalyst. Also, the probable mechanism of removal dye by photocatalytic function was studied using three types of scavengers of EDTA, Benzoic acid and benzoquinone to trap h^+^, ^•^OH and ^•^O_2_^−^ active specimens, respectively. Finally, it is found that ^•^OH and ^•^O_2_^−^ radicals promote photo-degradation of dye.

## CRediT authorship contribution statement

**Zeinab Talebzadeh:** Methodology, Investigation, Software, Formal analysis. **Maryam Masjedi-Arani:** Investigation, Writing – original draft, Writing - review & editing, Formal analysis. **Omid Amiri:** Visualization, Investigation, Software, Data curation. **Masoud Salavati-Niasari:** Visualization, Writing – original draft, Writing - review & editing, Validation, Investigation, Data curation, Conceptualization, Methodology, Supervision, Project administration.

## Declaration of Competing Interest

The authors declare that they have no known competing financial interests or personal relationships that could have appeared to influence the work reported in this paper.

## References

[1] Fujioka T., Ngo M.T.T., Makabe R., Ueyama T., Takeuchi H., Nga T.T.V., Bui X.-T., Tanaka H. (2021). Submerged nanofiltration without pre-treatment for direct advanced drinking water treatment. Chemosphere.

[2] Zhu Y., Wang W., Yu H., Wang A. (2020). Preparation of porous adsorbent via Pickering emulsion template for water treatment: A review. J. Environ. Sci..

[3] Ighalo J.O., Eletta O.A. (2020). Recent advances in the biosorption of pollutants by fish scales: a mini-review. Chem. Eng. Commun..

[4] Ranjeh M., Masjedi-Arani M., Salavati-Niasari M., Moayedi H. (2020). EDTA-modified sol-gel synthesis of monoclinic Li2MnO3 nanoparticles as an effective photocatalyst for degradation of organic dyes. J. Mol. Liq..

[5] Habibi-Yangjeh A., Asadzadeh-Khaneghah S., Feizpoor S., Rouhi A. (2020). Review on heterogeneous photocatalytic disinfection of waterborne, airborne, and foodborne viruses: Can we win against pathogenic viruses?. J. Colloid Interface Sci..

[6] Saravanan A., Kumar P.S., Vo D.-V.-N., Yaashikaa P.R., Karishma S., Jeevanantham S., Gayathri B., Bharathi V.D. (2020). Photocatalysis for removal of environmental pollutants and fuel production: a review. Environ. Chem. Lett..

[7] Karami M., Ghanbari M., Amiri O., Salavati-Niasari M. (2020). Enhanced antibacterial activity and photocatalytic degradation of organic dyes under visible light using cesium lead iodide perovskite nanostructures prepared by hydrothermal method. Sep. Purif. Technol..

[8] Orooji Y., Ghanbari M., Amiri O., Salavati-Niasari M. (2020). Facile fabrication of silver iodide/graphitic carbon nitride nanocomposites by notable photo-catalytic performance through sunlight and antimicrobial activity. J. Hazard. Mater..

[9] Asadzadeh-Khaneghah S., Habibi-Yangjeh A. (2020). g-C3N4/carbon dot-based nanocomposites serve as efficacious photocatalysts for environmental purification and energy generation: A review. J. Cleaner Prod..

[10] Hitam C., Jalil A. (2020). A review on exploration of Fe2O3 photocatalyst towards degradation of dyes and organic contaminants. J. Environ. Manage..

[11] Ghanbari M., Salavati-Niasari M. (2018). Tl4CdI6 nanostructures: facile sonochemical synthesis and photocatalytic activity for removal of organic dyes. Inorg. Chem..

[12] Tao L., Huang J., Dastan D., Wang T., Li J., Yin X., Wang Q. (2020). CO2 capture and separation on charge-modulated calcite. Appl. Surf. Sci..

[13] Sayadi M.H., Homaeigohar S., Rezaei A., Shekari H. (2021). Bi/SnO 2/TiO 2-graphene nanocomposite photocatalyst for solar visible light–induced photodegradation of pentachlorophenol. Environ. Sci. Pollut. Res..

[14] Mandal B., Panda J., Paul P.K., Sarkar R., Tudu B. (2020). MnFe2O4 decorated reduced graphene oxide heterostructures: nanophotocatalyst for methylene blue dye degradation. Vacuum.

[15] Aadil M., Rahman A., Zulfiqar S., Alsafari I.A., Shahid M., Shakir I., Agboola P.O., Haider S., Warsi M.F. (2021). Facile synthesis of binary metal substituted copper oxide as a solar light driven photocatalyst and antibacterial substitute. Adv. Powder Technol..

[16] Zhao W., Wei Z., Zhang X., Ding M., Huang S., Yang S. (2020). Magnetic recyclable MnFe2O4/CeO2/SnS2 ternary nano-photocatalyst for photo-Fenton degradation. Appl. Catal. A.

[17] Zeng J., Wang H., Zhang Y., Zhu M.K., Yan H. (2007). Hydrothermal synthesis and photocatalytic properties of pyrochlore La2Sn2O7 nanocubes. J. Phys. Chem. C.

[18] Quader A., Mustafa G.M., Abbas S.K., Ahmad H., Riaz S., Naseem S., Atiq S. (2020). Efficient energy storage and fast switching capabilities in Nd-substituted La2Sn2O7 pyrochlores. Chem. Eng. J..

[19] Fathinezhad M., AbbasiTarighat M., Dastan D. (2020). Chemometrics heavy metal content clusters using electrochemical data of modified carbon paste electrode. Environ. Nanotechnol. Monit. Manage..

[20] Feng X., Liu R., Xu X., Tong Y., Zhang S., He J., Xu J., Fang X., Wang X. (2021). Stable CuO/La2Sn2O7 catalysts for soot combustion: Study on the monolayer dispersion behavior of CuO over a La2Sn2O7 pyrochlore support. Chin. J. Catal..

[21] Haghnegahdar N., Abbasi Tarighat M., Dastan D. (2021). Curcumin-functionalized nanocomposite AgNPs/SDS/MWCNTs for electrocatalytic simultaneous determination of dopamine, uric acid, and guanine in co-existence of ascorbic acid by glassy carbon electrode. J. Mater. Sci.: Mater. Electron..

[22] Wang S.M., Xiu Z.L., Lü M.K., Zhang A.Y., Zhou Y.Y., Yang Z.S. (2007). Combustion synthesis and luminescent properties of Dy3+-doped La2Sn2O7 nanocrystals. Mater. Sci. Eng., B.

[23] Chen S., Pan B., Zeng L., Luo S., Wang X., Su W. (2017). La 2 Sn 2 O 7 enhanced photocatalytic CO 2 reduction with H 2 O by deposition of Au co-catalyst. RSC Adv..

[24] Mehdizadeh P., Masjedi-Arani M., Salavati-Niasari M. (2021). Green solid-state fabrication of new nanocomposites based on La–Fe–O nanostructures for electrochemical hydrogen storage application. Int. J. Hydrogen Energy.

[25] Shan K.e., Yi Z.-Z., Yin X.-T., Cui L., Dastan D., Garmestani H., Alamgir F.M. (2021). Diffusion kinetics mechanism of oxygen ion in dense diffusion barrier limiting current oxygen sensors. J. Alloy. Compd..

[26] Tao L., Huang J., Dastan D., Wang T., Li J., Yin X., Wang Q. (2021). New insight into absorption characteristics of CO2 on the surface of calcite, dolomite, and magnesite. Appl. Surf. Sci..

[27] Masjedi-Arani M., Salavati-Niasari M. (2016). A simple sonochemical approach for synthesis and characterization of Zn2SiO4 nanostructures. Ultrason. Sonochem..

[28] Masjedi-Arani M., Ghiyasiyan-Arani M., Amiri O., Salavati-Niasari M. (2020). CdSnO3-graphene nanocomposites: Ultrasonic synthesis using glucose as capping agent and characterization for electrochemical hydrogen storage. Ultrason. Sonochem..

[29] Masjedi-Arani M., Salavati-Niasari M. (2018). Cd2SiO4/Graphene nanocomposite: Ultrasonic assisted synthesis, characterization and electrochemical hydrogen storage application. Ultrason. Sonochem..

[30] Peng Y., Xia C., Cui M., Yao Z., Yi X. (2021). Effect of reaction condition on microstructure and properties of (NiCuZn) Fe2O4 nanoparticles synthesized via co-precipitation with ultrasonic irradiation. Ultrason. Sonochem..

[31] Khoobchandani M., Zambre A., Katti K., Lin C.-H., Katti K.V. (2013). Green nanotechnology from brassicaceae: development of broccoli phytochemicals–encapsulated gold nanoparticles and their applications in nanomedicine. Int. J. Green Nanotechnol..

[32] Latté K.P., Appel K.-E., Lampen A. (2011). Health benefits and possible risks of broccoli–An overview. Food Chem. Toxicol..

[33] Osuntokun J., Onwudiwe D.C., Ebenso E.E. (2018). Aqueous extract of broccoli mediated synthesis of CaO nanoparticles and its application in the photocatalytic degradation of bromocrescol green. IET Nanobiotechnol..

[34] Zhao Z., Sun Y., Dong F. (2015). Graphitic carbon nitride based nanocomposites: a review. Nanoscale.

[35] Akhundi A., Badiei A., Ziarani G.M., Habibi-Yangjeh A., Muñoz-Batista M.J., Luque R. (2020). Graphitic carbon nitride-based photocatalysts: toward efficient organic transformation for value-added chemicals production. Mol. Catal..

[36] Hasija V., Nguyen V.-H., Kumar A., Raizada P., Krishnan V., Khan A.A.P., Singh P., Lichtfouse E., Wang C., Thi Huong P. (2021). Advanced activation of persulfate by polymeric g-C3N4 based photocatalysts for environmental remediation: A review. J. Hazard. Mater..

[37] Masjedi-Arani M., Salavati-Niasari M. (2017). Simple size-controlled fabrication of Zn2SnO4 nanostructures and study of their behavior in dye sensitized solar cells. Int. J. Hydrogen Energy.

[38] Tauc J., Grigorovici R., Vancu A. (1966). Optical properties and electronic structure of amorphous germanium. Phys. Status Solidi (b).

[39] Fu Z., Yang H.K., Moon B.K., Choi B.C., Jeong J.H. (2009). Synthesis, characterization and luminescence properties of Eu3+-doped La2Sn2O7 nanospheres. Curr. Appl Phys..

[40] Leofanti G., Padovan M., Tozzola G., Venturelli B. (1998). Surface area and pore texture of catalysts. Catal. Today.

[41] Abdolhoseinzadeh A., Sheibani S. (2020). Enhanced photocatalytic performance of Cu2O nano-photocatalyst powder modified by ball milling and ZnO. Adv. Powder Technol..

[42] Wang J., Wang G., Cheng B., Yu J., Fan J. (2021). Sulfur-doped g-C3N4/TiO2 S-scheme heterojunction photocatalyst for Congo Red photodegradation. Chin. J. Catal..

[43] Li J., Zhang M., Li Q., Yang J. (2017). Enhanced visible light activity on direct contact Z-scheme g-C3N4-TiO2 photocatalyst. Appl. Surf. Sci..

[44] Mahdiani M., Soofivand F., Ansari F., Salavati-Niasari M. (2018). Grafting of CuFe12O19 nanoparticles on CNT and graphene: eco-friendly synthesis, characterization and photocatalytic activity. J. Cleaner Prod..

